# Cell and rat serum, urine and tissue metabolomics analysis elucidates the key pathway changes associated with chronic nephropathy and reveals the mechanism of action of rhein

**DOI:** 10.1186/s13020-023-00862-1

**Published:** 2023-12-01

**Authors:** Li Wang, Xixi Yu, Hongju Li, Dahong He, Su Zeng, Zheng Xiang

**Affiliations:** 1 Department of Pharmaceutical Analysis and Drug Metabolism, College of Pharmaceutical Sciences, Zhejiang University, Hangzhou, 310058 Zhejiang People’s Republic of China; 2 Medical School, Hangzhou City University, Hangzhou, 310015 China; 3 Key Laboratory of Novel Targets and Drug Study for Neural Repair of Zhejiang Province, School of Medicine, Hangzhou City University, Hangzhou, 310015 China; 4 School of Pharmaceutical Sciences, Wenzhou Medical University, Wenzhou, 325035 China; 5 College of Life Sciences and Medicine, Zhejiang Sci-Tech University, Hangzhou, 310018 China

**Keywords:** Rhein, Chronic kidney disease, Metabolomics, Fibrosis, UPLC-QTOF-MS

## Abstract

**Background:**

Rhein can significantly delay the progression of chronic nephropathy. However, its mechanism of action has not been adequately elaborated, which hinders its extensive clinical application. In this work, the effects of rhein on models of TGF-β-induced NRK-49F cellular fibrosis and rat renal ischemia–reperfusion fibrosis were evaluated using metabolomics and western blotting.

**Methods:**

The metabolic profiles of NRK-49F cells and rat urine, serum, and kidney tissues in the control, model, and rhein groups were investigated using UPLC-QTOF-MS. The levels of p-P65, p-IKK, p-AKT, p-P38, p-JNK and AP-1 in NRK-49F cells were measured using western blotting and immunofluorescence methods. Molecular docking and network pharmacology methods were employed to explore the relationship between the potential targets of rhein and key proteins in the NF-κB and MAPK signaling pathways.

**Results:**

Various potential metabolites, including sphingolipids, ceramides, phosphatidylcholine, and lysophosphatidylcholine,14-hydroxy-E4-neuroprostane E, and 5-HPETE, were present in the cell, tissue, urine, and serum samples; however, few metabolites matches exactly among the four type of biological samples. These differential metabolites can effectively differentiated between the control, model, and rhein groups. Pathway enrichment analysis of differential metabolites unveiled that sphingolipid metabolism, arachidonic acid metabolism, and glycerophospholipid metabolism were closely related to nephropathy. Phosphorylation levels of AKT, IKK, P65 and AP-1 in NRK-49F cells was reduced by rhein treatment. Network pharmacology and molecular docking showed that the potential targets of rhein might regulated the expression of MAPK and AKT in the NF-κB and MAPK signaling pathways.

**Conclusion:**

In brief, rhein might delays the progression of chronic nephropathy via the metabolic pathways, NF-κB and MAPKs signaling pathways, which provides the foundation for its development and clinical application.

**Supplementary Information:**

The online version contains supplementary material available at 10.1186/s13020-023-00862-1.

## Introduction

Chronic kidney disease (CKD) is a significant global public health concern. It is not only a common disease in the elderly and obese but a major complication of diabetes. Treatments for end-stage renal disease (e.g., peritoneal dialysis and haemodialysis) and kidney transplantation have resulted in a huge economic burden [1]. As CKD progresses, patients are at an increased risk of cardiovascular complications, mineral and bone disorders, infections, acute kidney injury, and death. The probability of death is at least 8–10 times higher in patients with CKD than in healthy individuals. Furthermore, the incidence and prevalence of CKD continue to rise [2]. Renal fibrosis, common in all types of CKD that progress to the end stage, is a complex and progressive pathophysiological process. Many signaling pathways, such as integrin/ILK, TGF-β/Smad, p38MAPK, HIF, NF-κB, and Wnt/β-catenin are directly or indirectly involved in the pathophysiological process of renal fibrosis (RF) [3–7].

Rhein is extracted and isolated from the rhizomes of several traditional medicinal plants, such as rhubarb, aloe, senna leaf, and *Polygoni Multiflori* Radix [8]. It has various pharmacological activities and significant anti-inflammatory and anti-fibrotic effects. Tu et al. [9] observed that rhein can inhibits the autophagy in rat renal tubular cells through regulating the AMPK-dependent mTOR pathway and important proteins in the ERK and p38-MAPK signaling pathways, thereby delaying the progression of RF. Rhein exerts immunoregulatory and anti-inflammatory effects by inhibiting the expression and phosphorylation of proteins related to the NF-κB signaling pathway, reducing the activation of NF-κB, and hindering the transcription of NF, thereby exerting renal protective effects [10]. Additionally, rhein protects endothelial cells from H_2_O_2_-induced oxidative damage [11], significantly reduces the malondialdehyde (MDA) content in cerebral ischemia–reperfusion injury model rats, and significantly increases the activities of superoxide dismutase (SOD), glutathione peroxidase (GSH-Px), and catalase (CAT), thereby hindering oxidative stress and exerting renoprotective effect [12]. In our studies of Bushen Huoxue Formula [13] and rhubarb [14] for the treatment of CKD, rhein was identified as one of the main active components; however, its mechanism of action in delaying CKD has not been fully elucidated.

Metabolomics enables the identification and quantification of all endogenous small molecules in a given sample [15], with the potential to detect hundreds to thousands of metabolites in each sample [16]. This approach can be used to explore the metabolic changes in disease states and under the action of drugs at the molecular level. For example, van der Kloet et al. [17] used GC–MS and LC–MS to detect the urine samples of patients with diabetic nephropathy, revealing the roles of acylcarnitine, acylglycine, and metabolites related to tryptophan metabolism. Kobayashi et al. [18] used GC–MS and LC–MS for the metabolomic analysis of serum samples from patients with CKD and used differential metabolites to construct a multiple regression equation to predict the CKD stage based on cystatin C-based eGFR with 81.3% accuracy. Moreover, Chen [19] and Hu et al. [20] analyzed the serum of patients with CKD using a metabolomic approach and screened out some metabolites, such as taurine, 5-methoxytryptophan, tiglylcarnitine, canavaninosuccinate, acetylcarnitine, 5,6-dihydroxyeicosatrienoic acid, 5-hydroxyeicosatetraenoic acid, and 9(10)-epoxyoctadecenoic acid. Although many metabolomics analyses have focused on the mechanism underlying kidney disease, the mechanisms of action of drugs, and the identification and screening of differential metabolites for RIF have not been effective [21], with substantial variation in results among studies and a narrow focus.

In this study, metabolomics using UPLC-QTOF-MS was used to explore the mechanism by which rhein delays chronic nephropathy at the metabolic level. The types and contents of differential metabolites in different types of samples and groups (i.e., a control, model, and rhein groups) were evaluated to provide the basis for the clinical application and new drug development of rhein.

## Methods

### Chemical reagents and antibodies

Rhein (purity > 98%, Batch No. DD0029) was purchased from Chengdu Desite Bio-Technology Co., Ltd. (Sichuan, China). Transforming growth factor-β1 with the purity ≥ 98% was purchased from Sigma-Aldrich (St. Louis, MO, USA). Kits for quantifying serum creatinine (Scr) and blood urea nitrogen (BUN) were purchased from the Nanjing Jiancheng Bioengineering Research Institute (Nanjing, China). HPLC-grade trichloromethane, acetonitrile, and methanol were obtained from Merck (Darmstadt, Germany). Trypsin digestive juice, streptomycin mixture solution, high-glucose DMEM, phosphate buffer (PBS), and foetal bovine serum (FBS) were obtained from Gibco (USA); 4% paraformaldehyde fixative, Triton X-100, and DAPI were purchased from Solarbio, China; α-SMA (14395-1-AP), FN (15613-1-AP), Collagen I (No. AB34710), GAPDH (10494-1-AP), JNK (No. 24164-1-AP), P38 (No. 14064-1-AP), P65 (No. 14220-1-AP), IKK (No. 15649-1-AP), and AP-1 (No.22114-1-AP) antibodies were obtained from Proteintech, China; AKT (#75692) and p-AKT (#9018) antibodies were obtained from Cell Signaling Technology, USA; p-JNK (No.AF3318), p-P38 (No.AF4001), p-IKK (No.AF3013), and p-P65 (No.AF2006) antibodies were obtained from Affinity, China; horseradish peroxidase-conjugated secondary antibodies and fluorescent dye-conjugated secondary antibodies were obtained from Proteintech, China; and the BCA kit was obtained from Thermo Fisher, USA. Dimethyl sulfoxide (DMSO) was obtained from Solarbio (China) and bovine serum albumin (BSA) was obtained from Dalian Meilun Biotechnology Co., Ltd. (China).

### Animals

SD rats (male, n = 18, 200–220 g) were purchased by the Laboratory Animal Centre of Wenzhou Medical University (ethical approval number: wydw2021-0720). Rats in each group were housed in the same cage at 25 °C with a humidity of approximately 50% and was adapted for 1 week in a well-ventilated environment, during which they were provided free access to food and water. They were randomly divided into three groups (6 rats each): sham operation group, ischemia–reperfusion injury group, and rhein group. Based on our previous work [22–24], the dose level of rhein was 120 mg/kg, prepared using 0.5% sodium carboxymethyl cellulose (CMC-Na). The sham operation and model groups were daily treated with the same volume of blank CMC-Na solution.

### Effect of TGF-β on cells and determination of the concentration of rhein

Cells in the experiments were cultured in high-glucose DMEM containing 10% FBS and 1% penicillin–streptomycin mixture and placed in a constant temperature cell incubator under 5% CO_2_, saturated humidity and at 37 °C. Cells in the logarithmic growth phase were digested with trypsin and inoculated at 2 × 10^5^ cells/mL into a 6-well culture plate. After 24 h of culture, high-glucose DMEM containing 1% FBS was used to starve the cells overnight. Control and 10 ng/mL TGF-β1 induction groups. The rhein concentrations in the administration groups were 1, 5, 10, and 20 ng/mL. After cells were cultured for 48 h, they were washed twice with pre-cooled PBS. The NP-40 cell lysate was added, and the cells were lysed in an ice bath at 4 °C for 30 min. Cells were subsequently collected into 2.0 mL EP tubes using a cell scraper, and the cell supernatant was taken after centrifugation at 12,500*g* for 8 min at 4 °C. Then, 10 μL of the supernatant was taken out and the protein content was determined using the BCA method. The remaining supernatant was added to 4 × SDS loading buffer, denatured by heating, and separated using 10% SDS-PAGE. After blocking the membrane with skim milk for 1.5 h, the membrane was washed twice and incubated with the specific primary antibodies for 12 h at 4 °C. After washing twice, the membrane was incubated with horseradish peroxidase-conjugated secondary antibodies, and the concentration of rhein was determined according to the observed band.

### Cell sample preparation

Cells in the logarithmic growth phase were treated with trypsin and then inoculated into 6-well culture plates at 7.5 × 10^5^ cells/mL. After 24 h, cells were synchronized by overnight starvation in high-glucose DMEM containing 1% FBS. After synchronization, the cells were divided into three groups (n = 6/group), namely the control group, 10 ng/mL TGF-β1 induction group, and rhein administration group, and were treated under 5% CO_2_ for 48 h and at 37 °C.

For the metabolomics experiments, after 48 h, the medium was quickly discarded, and cells were washed with 1 mL of cold PBS three times. Immediately after washing, the cells were quenched with liquid nitrogen to prevent continued metabolism. After the liquid nitrogen was evaporated, 1 mL of pre-cooled cell extract was added (water: methanol: chloroform = 1:9:1), and the cells were collected using a scraper. Cells were treated in liquid nitrogen for 3 min and thawed at 25 °C. This process was repeated five times to disrupt cells, dissolve cellular metabolites, and precipitate proteins. The cell supernatant was collected after centrifugation at 12,500*g* for 15 min at 4 °C and dried with nitrogen. Before injection, the samples were reconstituted in 300 μL of methanol solution, vortexed, and centrifuged, and 60 μL of the supernatant was injected into UPLC-QTOF-MS.

For pharmacological experiments, after treating the cells for 48 h, the cells were washed three times with 1 mL of pre-cooled PBS, 100 μL of NP-40 lysate was added, and cells were lysed in a 4 °C ice bath for 30 min and scraped off with a cell scraper. The scraped cells were centrifuged at 12,000*g* for 5 min at 4 °C. Then, the supernatant, which contained the total protein in the sample, was aspirated. Immunoreactive bands were measured after incubation with horseradish peroxidase-conjugated secondary antibodies using an enhanced chemiluminescence reagent (Bio-Rad). ImageJ was used for protein quantification and normalization to the respective controls. GAPDH was used to quantify cytoplasmic proteins, and total protein was used to quantify phosphorylated proteins.

### Immunofluorescence

NRK-49F cells in the logarithmic phase were inoculated onto coverslips at a density of 50,000 cells/mL. After treatment for 48 h, the cells were fixed with 4% paraformaldehyde for 30 min, permeabilized with 1% Triton X-100 in an ice bath for 30 min, blocked with 3% BSA at room temperature for 2 h, and incubated with specific primary antibodies at 4 °C for 12 h. The cells were then treated with fluorescent dye-conjugated secondary antibodies at room temperature for 2 h. Finally, the cells were counterstained with DAPI. Imaging was performed using an upright fluorescence microscope.

### Sample collection

Urine excreted by rats 12 h before unilateral ischemia–reperfusion injury (UIRI) modeling, after UIRI modeling, and on the 14th day of rhein administration was collected in metabolic cages. After centrifugation at 12,000*g* for 10 min to precipitate impurities, the supernatant was taken and stored at − 80 °C for urine metabolomics. After the samples were thawed at 4 °C, acetonitrile was added at a ratio of sample: acetonitrile = 1:1, vortexed for 3 min, and centrifuged at 12,000*g* for 10 min. The supernatant was used for testing.

For serum collection, 1–1.5 mL of tail vein blood was collected in EP tubes before UIRI modeling, after modeling, and on days 3, 7, and 14 after rhein administration. The upper serum layer was taken after centrifugation at 3000 rpm for 10 min, divided into three parts, and stored at − 80 °C for the determination of Scr and BUN levels and detection of endogenous metabolites in serum samples. The samples were thawed at 4 °C, and acetonitrile was added at a volume ratio of sample: acetonitrile = 1:3. The samples were vortexed for 3 min, centrifuged at 12,000*g* for 15 min, and the supernatants were collected for testing.

For tissue collection, rats in each group were anesthetized using 10% chloral hydrate on day 14, and cardiac perfusion was performed. The right kidney was removed and divided longitudinally into two parts. Then one part was fixed in paraformaldehyde for subsequent pathological examination and the other was stored in a − 80 °C for subsequent tissue metabolomic assays. After each sample (0.1 g) was thawed at 4 °C, 800 μL of acetonitrile was put in 1.5 mL EP tube. The samples were homogenized for 6 min, centrifuged at 12,000*g* for 15 min, and the supernatants were collected for testing.

### Determination of biochemical indexes and pathological changes

Serum samples stored in a − 80 °C refrigerator were thawed at 4 °C. Serum creatinine and BUN levels were determined using the manufacturer’s instructions. Hematoxylin–eosin (HE) and Masson staining were performed on kidney tissues after embedding and sectioning. The pathological status of the kidney tissues after UIRI modeling was observed using an upright fluorescence microscope.

### UPLC-QTOF-MS conditions

The UPLC-QTOF-MS system consisted of an Acquity ultra-performance liquid chromatograph and a Xevo G2-XS QTof quadrupole tandem time-of-flight mass spectrometer (Waters Corporation, Milford, MA, USA). All samples were separated using HSS T3 C18 column (2.1 × 100 mm, 1.8 μm) (Waters Corporation). The mobile phases consisted of 0.1% aqueous formic acid (solvent A) and acetonitrile (solvent B), and the program of gradient elution was as follows: 0–0.5 min, 5% B; 0.5–20.5 min, 5–95% B, 20.5–25 min, 5% B. The flow rate was 0.3 mL/min, the column temperature was 30 °C, the injection volume was 2 μL, and the injector temperature was 10 °C.

The mass spectrometer used MS^E^ continuum mode, and ESI with both positive and negative ion modes were detected, capillary voltage was 2.5 kV, cone voltage was 50 V, ion source temperature was 100 °C, the gas in the cone hole was nitrogen and at a flow rate of 50 L/h, the desolvation gas was nitrogen at a flow rate of 800 L/h, the temperature of the desolvation gas was 400 °C, scan time was 0.2 s, scan interval was 0.015 s, low-channel collision energy was 6 eV, high-channel collision energy was 20–30 eV, and mass range was 50–1200 m*/z*.

Leucine-enkephalin (5 ng/mL) was used as the calibration solution to determine whether the axis of the mass spectrum was biased. In addition, 10 μL of each sample was mixed to prepare quality control samples (QCs) to evaluate the precision and repeatability of the method. Before sample analysis, 3–5 QCs were continuously tested, and a QC sample was inserted after every 10 samples.

### Data processing method

The raw data for each sample obtained by UPLC-QTOF-MS were imported into Progenesis QI ver.2.2 (Nonlinear Dynamic) for peak alignment, deconvolution, and normalization. The molecular structures of the detected metabolites were identified and confirmed based on the consistency of their MS and MS/MS data with those of the metabolites in the HMDB, METLIN, and LIPD MAPS databases. The errors of MS and MS/MS values were set at < 0.1 and 0.5 Da, respectively. To verify the reliability of the obtained data, using the Noreva 2.0 website (http://47.99.36.124/noreva/), the data were pre-processed according to the QC samples as follows. Metabolites with more than 80% missing values in the samples were deleted, the missing values were supplied using the k-nearest neighbour algorithm, the RSD of the QC was < 30%, and a local polynomial fit was performed.

The reliability and precision of UPLC-QTOF-MS method were verified through duplicate analyses of 6 injections of the same QCs and 6 parallel samples. The RSD of the peak retention times and areas were < 5.0%. The precision and reliability of the proposed cell, serum, serum, and tissue methods were satisfactory for metabolomics.

Multivariate statistical methods, including a principal component analysis (PCA), partial least squares discriminant analysis (PLS-DA), and orthogonal partial least squares discriminant analysis (OPLS-DA), were performed on the pre-processed data using SIMICA (version 14.0, Umetrics AB, Sweden). Differential metabolites were screened based on the variable importance in projection (VIP) in the loading map (≥ 1.0). One-way ANOVA (*P* = 0.05) was evaluated using IBM SPSS (version 22.0, IBM Corporation, Armonk, NY, USA), and differential metabolites were screened using fold change (FC) > 2 or < 0.5 as thresholds. Pathway enrichment was evaluated using the MetaboAnalyst 5.0 platform. The obtained data are expressed as mean ± standard deviation (x ± s).

### Network pharmacology and molecular docking of rhein

The STITCH database was employed to identify potential targets of rhein with a high confidence level of 0.7. Rhein was docked to potential targets using AutoDock Vina. The crystal structures were obtained from the Protein Data Bank. The mean values and standard deviations were obtained through the calculations of three molecular docking. PYMOL was performed to visualize the processing of the molecular docking results. String database (Version:12.0, https://cn.string-db.org/) with a high confidence of 0.7 was used to search for interactions between the potential targets of rhein and proteins P65, IKK, AKT, P38, JNK, and AP-1.

## Results

### Serum creatinine and BUN contents

**Fig. 1:**
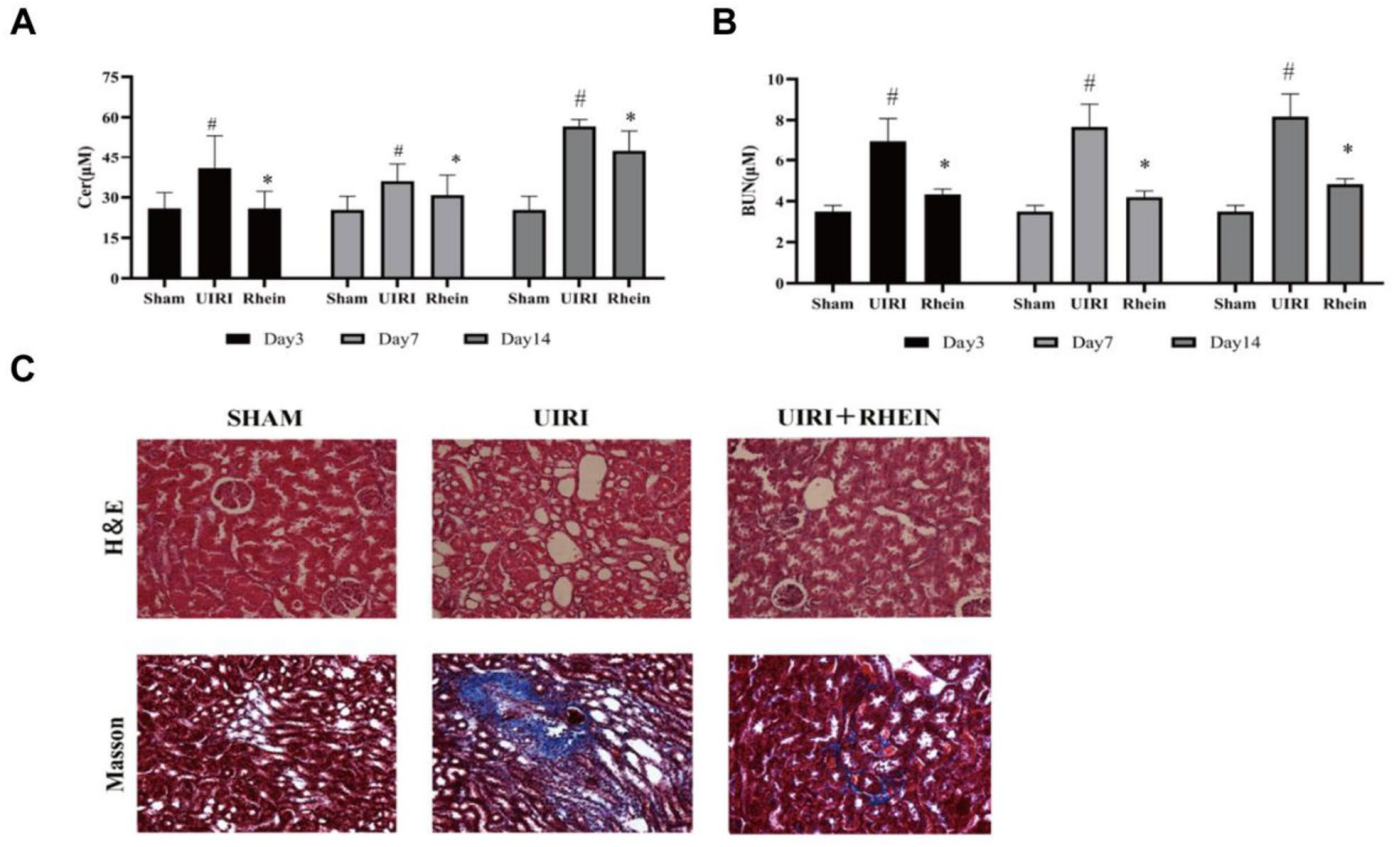
Biochemical indexes and HE and Masson pathological staining of rats. **A** Serum creatinine (CER) levels in rats in each group, compared with the sham group, *P < 0.05; compared with the UIRI group, ^#^*P* < 0.05; **B** blood urea nitrogen (BUN) levels in serum of rats in each group, compared with the sham group, *P < 0.05; compared with the UIRI group, ^#^*P* < 0.05; **C** histopathological results for each group

Scr levels were obviously higher in the UIRI group than in the sham group (*P* ≤ 0.05) and obviously lower in the rhein-treated group than in the UIRI group on days 3, 7, and 14 (*P* ≤ 0.05; Fig. 1A). The BUN level in the UIRI model group was obviously higher than that in the sham group (*P* ≤ 0.05) and significantly lower in the rhein-treated group than in the UIRI group on days 3, 7, and 14 (*P* ≤ 0.05; Fig. 1B). The significant increase in serum creatinine and BUN levels in the UIRI group indicated that the UIRI model was successfully established. Furthermore, serum creatinine and BUN levels decreased after rhein administration, suggesting that rhein delays the progression of chronic nephropathy.

### HE and Masson’s trichrome staining results

Renal interstitial widening, increased interstitial cells, collagen deposition, vacuoles, renal tubule lesions, glomerular atrophy, and renal interstitial fibrosis were observed in the UIRI group. Compared with those in the UIRI group, the vacuoles in the rhein administration group were reduced; collagen deposition, cell infiltration, and other changes in the UIRI group were attenuated, and the degree of fibrosis was reduced. The pathological results showed that rhein reduced the degree of renal interstitial damage (Fig. 1C).

#### Rhein inhibits cellular fibrosis induced by TGF-β

**Fig. 2:**
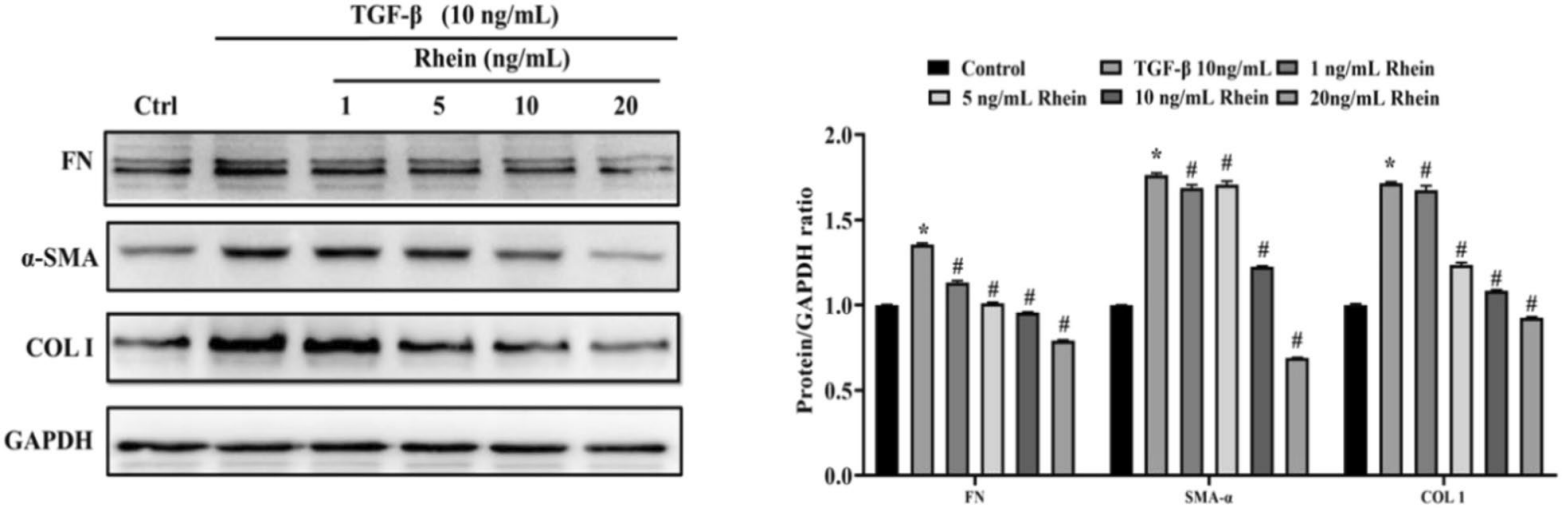
Expression of fibrosis-related factors in different groups of NRK-49F cells induced by 10 ng/mL TGF-β. Compared with the control group, *P < 0.05; compared with the 10 ng/mL TGF-β group, ^#^*P* < 0.05

When NRK-49F cells were induced by 10 ng/mL TGF-β, the protein expression contents of FN, α-SMA, and COL1 in the TGF-β group were significantly higher than those in the control group (*P* ≤ 0.05), and NRK-49F cell developed fibrosis. With the addition of rhein, the protein expression contents of FN, α-SMA, and COL1 were lower than those in the UIRI group (*P* ≤ 0.05). When the rhein concentration was increased to 10 ng/mL, both COL1 and FN protein expression levels significantly decreased (Fig. 2). Therefore, 10 ng/mL rhein was used for subsequent analysis of NRK-49F cells.

**Fig. 3:**
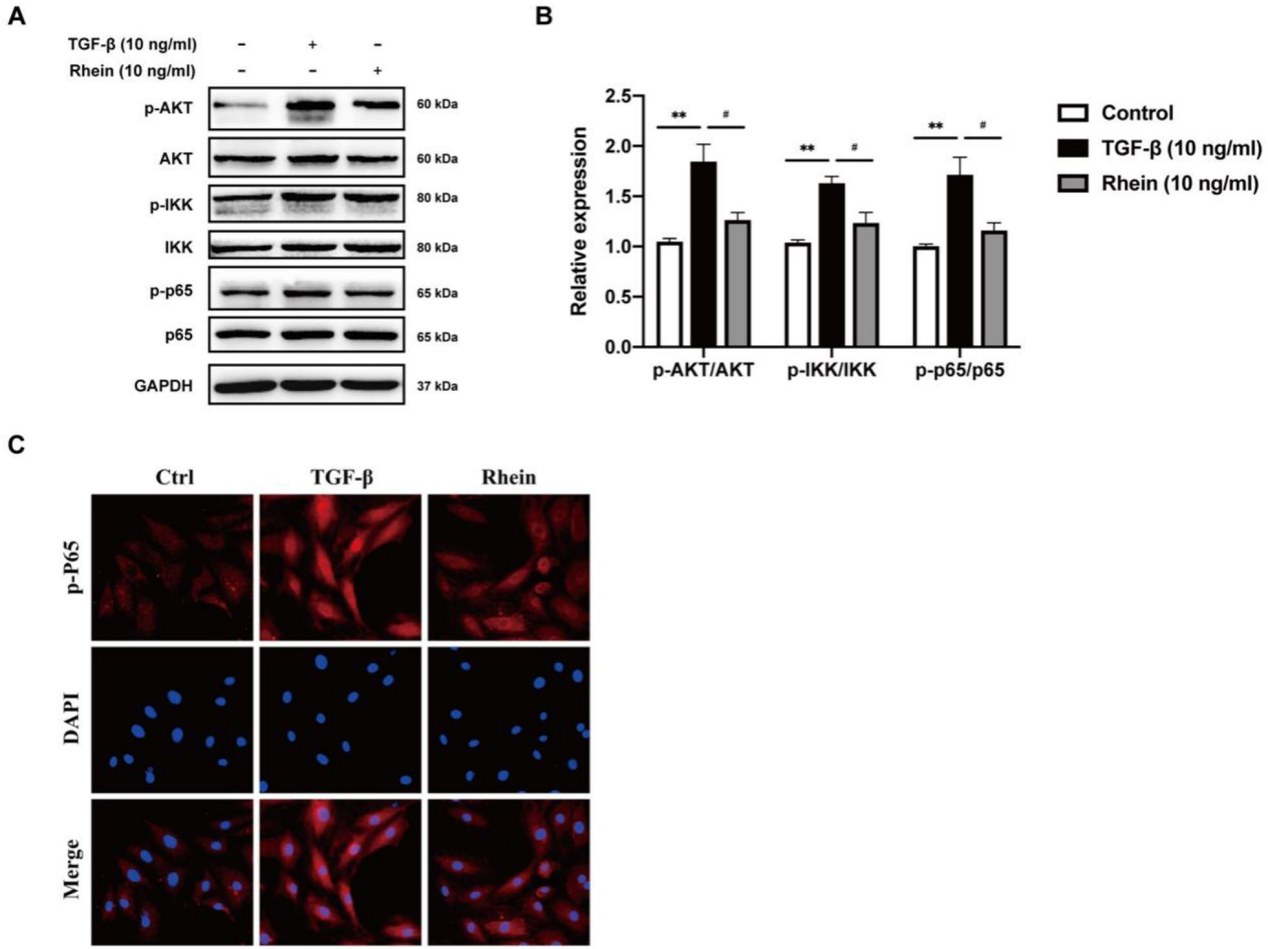
Rhein alleviates renal fibrosis through inhibition of NF-κB signaling pathway. **A** Western blot analysis of p-AKT, p-IKK, p-P65 in NRK-49F cells exposed to rhein and curcumin. **B** Quantification of p-P65/P65, p-IKK/IKK, p-AKT/AKT levels in NRK-49F cell. **C** Representative immunofluorescence images of p-P65. n = 3, Compared with the control group, ^*^P < 0.05, ^**^P < 0.01, ^***^ *P* < 0.001; compared with the 10 ng/mL TGF-β group, ^#^*P* < 0.05

Rhein significantly inhibits the activation of NF-κB and MAPK signaling pathways induced by TGF-β. The NF-κB signaling pathway involves biological processes such as immunity, stress response, inflammation, and apoptosis, and is closely related to the progression of RF. NF-κB is a pivotal protein in this pathway. Induction using 10 ng/mL TGF-β significantly increased AKT phosphorylation, thereby stimulating downstream IKK phosphorylation and activating NF-κB (P65) phosphorylation. Rhein (10 ng/mL) reduced the phosphorylation of AKT, IKK, and P65 and expression of p-Akt, p-IKK, and p-P65 (Fig. 3A and B). Immunofluorescence showed that after TGF-β stimulation, the phosphorylation of P65, the core protein in the NF-κB signaling pathway, and entry of p-P65 into cell nuclei increased significantly (Fig. 3C).

**Fig. 4:**
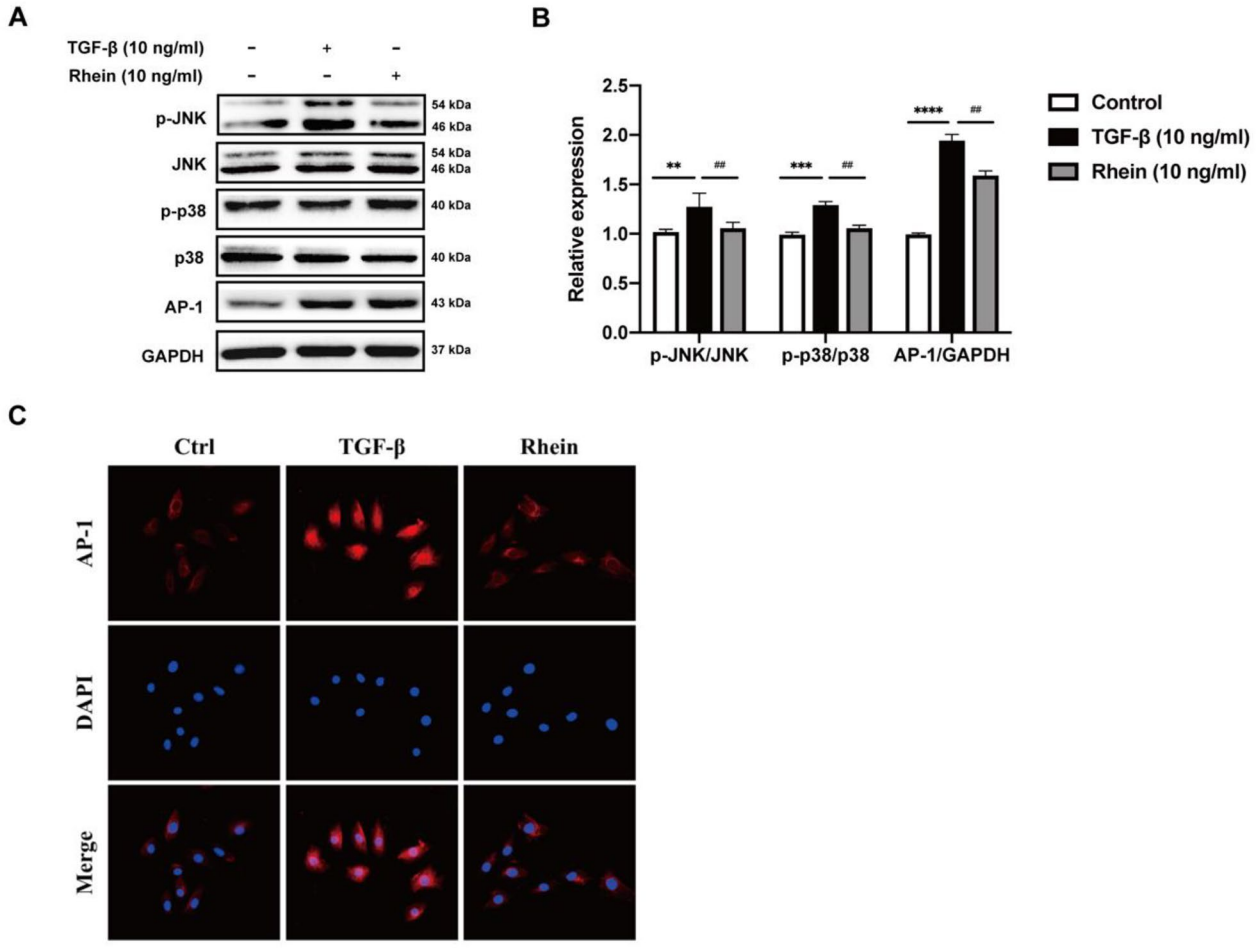
Rhein alleviates renal fibrosis through inhibition of MAPKs signaling pathway. **A** Western blot analysis of p-JNK, JNK, p-P38, P38 and AP-1 in NRK-49F cells exposed to rhein. **B** Quantification of p-P38/P38, p-JNK/JNK, AP-1 levels in NRK-49F cell. **C** Representative Immunofluorescence images of AP-1. n = 3, Compared with the control group, ^*^P < 0.05, ^**^P < 0.01, ^***^ *P* < 0.001; compared with the 10 ng/mL TGF-β group, ^#^*P* < 0.05

The MAPK signaling pathway includes P38, JNK, and ERK, which play an important part in the occurrence and progression of inflammation. The progression of renal fibrosis is closely associated with inflammation. Induction using 10 ng/mL TGF-β significantly increased the phosphorylation of JNK and P38, resulting in an obvious increase in the expression of AP-1, a key downstream molecule (Fig. 4A and B). Immunofluorescence also showed that 10 ng/mL TGF-β obviously increased the expression of AP-1 in the cell and that rhein significantly decreased its expression (Fig. 4C).

### Cellular metabolomic analyses

**Fig. 5:**
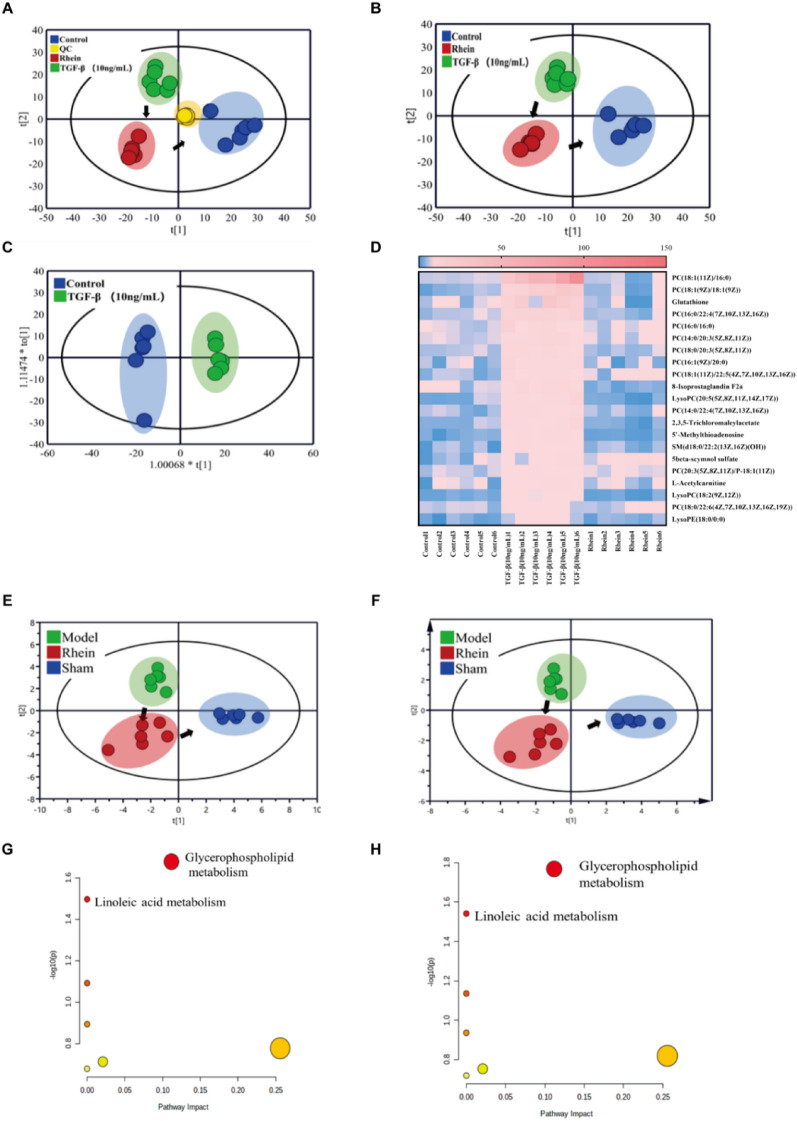
Metabolomics analysis of NRK-49F cells. **A** PCA score plot; **B** PLS score plot; **C** OPLA-DA score plots of control and TGF-β induction groups; **D** heatmap of relative peak areas of differential metabolites in different groups. PCA (**E**) and PLS-DA (**F**) scores for different groups of cell samples based on differential metabolites. MetaboAnalyst pathway enrichment results based on 21 differential metabolites (**G**) and 20 differential metabolites (**H**)

The UPLC-QTOF-MS metabolic profiles of the different groups of NRK-49F cells are shown in Additional file 1: Figure S1. Progenesis QI identified 934 ion peaks in the positive ion mode. As shown in the PCA score plot in Fig. 5A, the QCs clustered together, indicating that the established metabolomics method has good reproducibility and precision. As shown in the PLS score plot in Fig. 5B, metabolic profiles differed significantly among the TGF-β1 induction, the control, and the rhein administration groups. The TGF-β1 induction group was clearly separated from the control group, suggesting that TGF-β1 successfully induced fibrosis in NRK-49F cells. The metabolic profile of the rhein administration group significantly deviated from that of the TGF-β1 induction group and approached that of the control group, suggesting that rhein could attenuate TGF-β1-induced changes in the metabolite profile. As shown in the OPLS-DA score plot in Fig. 5C, the TGF-β1 induction group was clearly distinguished from the control group, and the parameters of model evaluation, such as R^2^Y and Q^2^, were close to 1.0 (R^2^Y = 0.993 and Q^2^ = 0.913, respectively), indicating that the established model had good predictive ability. After treatment (Cell sample preparation), 21 differential metabolites associated with cellular fibrosis were identified (Table 1). Differential metabolites were increased in TGF-β1-induced cells, and 20 metabolites were recovered after rhein administration (Fig. 5D). PCA and PLS-DA for these differential metabolites (Fig. 5E and F) revealed similar changes in metabolic profiles to Fig. 5B, supporting the reliability of the screened differential metabolite classification.

**Table 1:** The potential cell metabolites in positive ion mode

No	Compound	VIP	P	FC	Model	Rhein
1	PC(18:1(11Z)/16:0)	7.08	1.00E−03	0.01	↑	↓
2	PC(18:1(9Z)/18:1(9Z))	3.39	1.00E−03	0.04	↑	↓
3	Glutathione	2.82	6.00E−03	0.17	↑	↓
4	LysoPE(18:0/0:0)	1.05	1.00E−03	0.25	↑	↓
5	PC(16:0/16:0)	1.96	1.00E−03	0.18	↑	↓
6	PC(14:0/20:3(5Z,8Z,11Z))	1.95	1.00E−03	0.21	↑	↓
7	PC(18:0/20:3(5Z,8Z,11Z))	1.78	1.00E−03	0.20	↑	↓
8	PC(16:1(9Z)/20:0)	1.60	3.00E−03	0.21	↑	↓
9	8-Isoprostaglandin F2a	1.55	1.00E−03	0.31	↑	↓
10	LysoPC(18:2(9Z,12Z))	1.09	1.00E−03	0.22	↑	↓
11	2,3,5-Trichloromaleylacetate	1.40	1.00E−03	0.20	↑	↓
12	5'-Methylthioadenosine	1.39	1.00E−03	0.18	↑	↓
13	5-beta-scymnol sulfate	1.20	3.00E−03	0.34	↑	↑
14	L-Acetylcarnitine	1.13	2.00E−03	0.37	↑	↓
15	PC(20:3(5Z,8Z,11Z)/P-18:1(11Z))	1.13	1.00E−03	0.43	↑	↓
16	PC(18:0/22:6(4Z,7Z,10Z,13Z,16Z,19Z))	1.07	3.00E−03	0.43	↑	↓
17	PC(14:0/22:4(7Z,10Z,13Z,16Z))	1.42	1.00E−03	0.26	↑	↓
18	PC(16:0/22:4(7Z,10Z,13Z,16Z))	2.79	1.00E−03	0.09	↑	↓
19	PC(18:1(11Z)/22:5(4Z,7Z,10Z,13Z,16Z))	1.57	1.00E−03	0.24	↑	↓
20	SM(d18:0/22:2(13Z,16Z)(OH))	1.30	1.00E−03	0.24	↑	↓
21	LysoPC(20:5(5Z,8Z,11Z,14Z,17Z))	1.51	2.00E−03	0.15	↑	↓

Differential metabolites were imported into the MetaboAnalyst platform for a metabolic pathway analysis. The differential metabolites were enriched in the linoleic acid pathways and glycerophospholipid pathways (Fig. 5G and H). Twenty differential metabolites recovered after rhein administration were still enriched in glycerophospholipid metabolism and linoleic acid metabolism.

### Metabolomics of urine samples

**Fig. 6:**
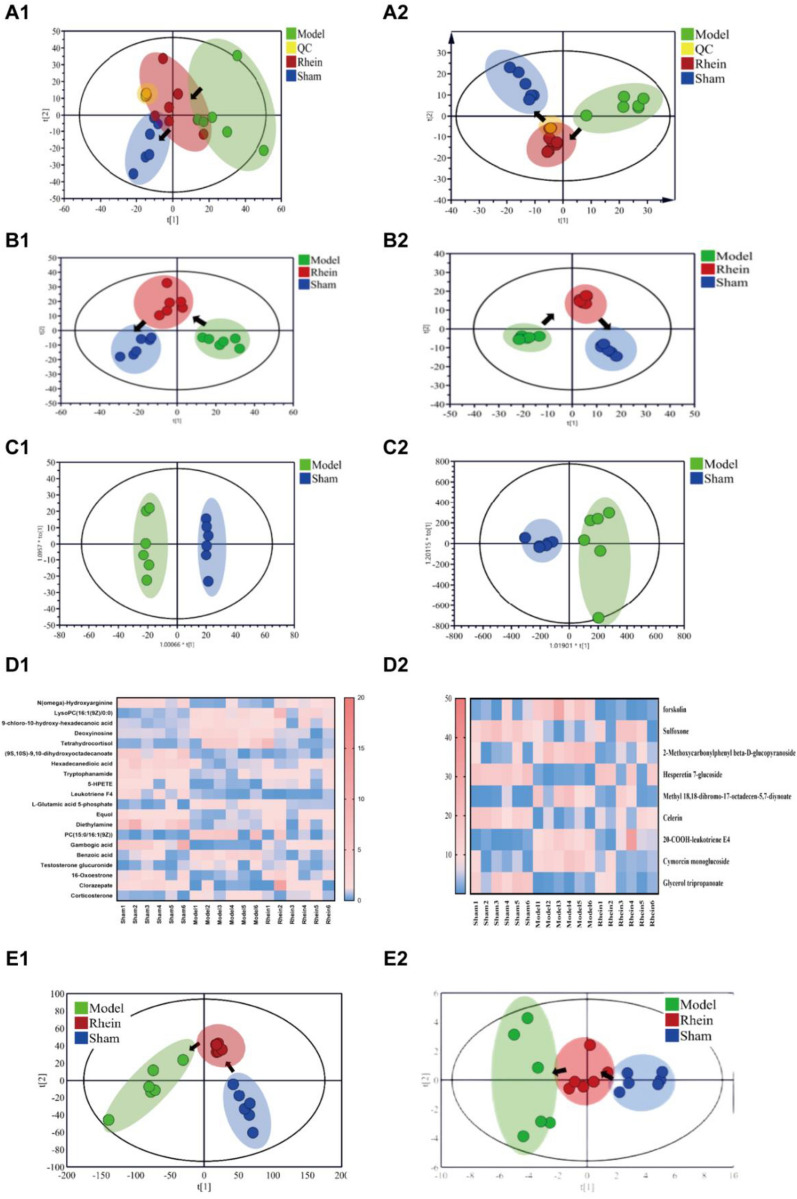
Metabolomics analysis of urine samples in the positive (1) and negative (2) model. **A** PCA score plot; **B** PLS score plot; **C** OPLA-DA score plots of control and TGF-β induction groups; **D** heatmap of relative peak areas of differential metabolites in different groups. PCA (**E1**) and PLS-DA (**E2**) scores for different groups of urine samples based on differential metabolites

Additional file 1: Figure S2 depicts the metabolic profiles of all samples in positive and negative ion modes. Using Progenesis QI, 2916 ion peaks in the positive ion mode and 2538 ion peaks in the negative ion mode were identified, respectively. As shown in the PCA score plots in positive and negative ion modes in Fig. 6A, the QCs were clustered well, indicating that the metabolomics method has good reproducibility and precision. As shown in the PLS score plots in Fig. 6B, the UIRI, the sham operation, and the rhein administration groups were significantly different, indicating that the metabolic profile in the urine of rats was altered significantly after modeling. The rhein administration group obviously deviated from the UIRI group and approached the sham operation group, indicating that rhein had effective anti-fibrotic effects. As shown in the OPLS-DA score plots in Fig. 6C, the UIRI group was obviously distinguished from the sham operation group, and the model evaluation parameters R^2^Y and Q^2^ were both close to 1.0 (in positive ion mode: R^2^Y = 0.997 and Q2 = 0.740; in negative ion mode: R2Y = 0.976 and Q2 = 0.836), indicating that the established model had a good predictive ability. According to the screening method described in Sect. 2.8, 20 differential metabolites in the positive ion mode and 9 differential metabolites in the negative ion mode were obtained (Table 2).

**Table 2:** The potential urine, serum and tissue metabolites in positive and negative mode

No	Compound	Ion mode	VIP	P	FC	Model	Rhein
Urine	*N*(omega)-Hydroxyarginine	+	1.87	2.56E−04	0.24	↓	↑
	LysoPC(16:1(9Z)/0:0)	+	1.86	3.17E−04	3.51	↑	↑
	9-chloro-10-hydroxy-hexadecanoic acid	+	1.82	1.23E−04	2.22	↑	↑
	Deoxyinosine	+	1.82	3.65E−04	2.1	↑	↓
	Tetrahydrocortisol	+	1.8	7.18E−03	5.7	↑	↓
	Hexadecanedioic acid	+	1.77	8.47E−04	0.35	↓	↑
	Tryptophanamide	+	1.74	1.02E−03	0.45	↓	↑
	5-HPETE	+	1.73	7.83E−04	0.28	↓	↑
	Leukotriene F4	+	1.66	2.27E−03	0.23	↓	↓
	l-Glutamic acid 5-phosphate	+	1.65	4.58E−03	3.64	↑	↓
	Equol	+	1.65	3.19E−03	0.49	↓	↑
	Diethylamine	+	1.63	4.99E−03	0.24	↓	↑
	PC(15:0/16:1(9Z))	+	1.62	6.34E−03	10.41	↑	↓
	Gambogic acid	+	1.62	4.42E−03	0.04	↓	↑
	Benzoic acid	+	1.61	1.61E−03	2.7	↑	↓
	Testosterone glucuronide	+	1.6	4.12E−02	2.16	↑	↓
	16-Oxoestrone	+	1.6	4.21E−03	0.25	↓	↑
	Clorazepate	+	1.6	3.82E−03	0.26	↓	↑
	Corticosterone	+	1.55	6.22E−03	2.16	↑	↓
	(9S,10S)-9,10-dihydroxyoctadecanoate	+	1.79	6.79E−03	0.29	↓	↓
	Forskolin	−	4.38	2.32E−02	380.46	↑	↓
	Sulfoxone	−	4.22	1.88E−02	0.02	↓	↑
	Hesperetin 7-glucoside	−	2.87	1.87E−02	16.94	↑	↓
	Celerin	−	2.02	3.50E−02	0.002	↓	↑
	20-COOH-leukotriene E4	−	1.69	3.70E−02	605.6	↑	↓
	Cymorcin monoglucoside	−	1.58	1.83E−02	97.87	↑	↓
	Glycerol tripropanoate	−	1.52	2.88E−02	0.03	↓	↑
	2-Methoxycarbonylphenyl beta-d-glucopyranoside	−	3.31	3.84E−02	27.99	↑	↓
	Methyl 18,18-dibromo-17-octadecen-5,7-diynoate	−	2.33	4.40E−02	72.2	↑	↓
Serum	Beta-d-3-Ribofuranosyluric acid	+	3.37	2.20E−02	0.05	↓	↑
	SM(d18:0/16:1(9Z))	+	3.1	3.34E−04	12.18	↑	↑
	PI(16:0/20:4(5Z,8Z,11Z,14Z))	+	2.99	1.86E−02	57.26	↑	↓
	Ceramide (d18:1/24:0)	+	2.88	3.02E−02	9.5	↑	↓
	PC(16:0/22:4(7Z,10Z,13Z,16Z))	+	2.33	1.34E−03	12.85	↑	↓
	Glycocholic acid	+	2.24	1.30E−02	0.05	↓	↓
	PC(16:0/16:0)	+	2.08	7.12E−05	6.22	↑	↓
	LysoPC(18:0)	+	1.92	8.61E−03	20.64	↑	↓
	(*S*)-Hydroxyoctanoyl-CoA	+	1.91	1.33E−02	5.62	↑	↓
	Norepinephrine	+	1.84	2.98E−02	3.65	↑	↑
	Oxidized glutathione	+	1.8	2.46E−02	0.06	↓	↑
	Palmitoyl glucuronide	+	1.74	1.10E−02	4.58	↑	↓
	Palmitaldehyde	+	1.72	1.11E−02	0.12	↓	↓
	PE(20:5(5Z,8Z,11Z,14Z,17Z)	+	1.61	2.91E−02	0.12	↓	↑
	/22:6(4Z,7Z,10Z,13Z,16Z,19Z))						
	PE(14:0/22:4(7Z,10Z,13Z,16Z))	−	3.2	5.17E−03	37.45	↑	↓
	DG(16:1(9Z)/20:1(11Z)/0:0)	−	2.85	5.60E−03	0.06	↓	↑
	SM(d18:0/24:0)	−	2.7	3.60E−02	0.04	↓	↓
	14-Hydroxy-E4-neuroprostane	−	2.69	4.60E−03	15.27	↑	↓
	Dihydrothymine	−	2.68	1.17E−03	28.65	↑	↓
Serum	Phenoxybenzamine	−	2.39	4.49E−03	0.05	↓	↓
	LysoSM(d18:1)	−	2.24	9.75E−03	6.48	↑	↓
	4-Acetamidobutanoic acid	−	1.72	3.42E−03	0.04	↓	↑
	*N*-Acetylornithine	−	1.66	1.42E−05	0.1	↓	↓
	Cer(d18:1/24:1(15Z))	−	1.64	1.70E−03	0.12	↓	↓
	PC(15:0/P-16:0)	−	1.63	2.35E−02	21.11	↑	↓
	PC(15:0/18:1(11Z))	−	1.62	4.37E−02	3.09	↑	↓
	2-Isopropylphenyl methylcarbamate	−	1.6	1.34E−03	0.07	↓	↓
	Dolichyl b-d-glucosylphosphate	−	1.6	5.13E−05	0.08	↓	↑
	Prostaglandin H2	−	1.57	4.66E−04	11.07	↑	↓
	10,11-epoxy-3,7,11-trimethyl-2E,6E-tridecadienoic acid	−	11.24	4.54E−02	0.02	↓	↑
	1-(4-Hydroxy-3-methoxyphenyl)-3-decanone	−	2.26	1.19E−02	3.78	↑	↓
	3-Hexaprenyl-4-hydroxy-5-methoxybenzoic acid	−	1.64	2.37E−06	0.08	↓	↑
Tissue	l-Phosphoarginine	+	2.76	6.70E−05	0.01	↓	↑
	1-Phosphatidyl-d-myo-inositol	+	2.44	6.31E−03	0.04	↓	↑
	15-Deoxy-d-12,14-PGJ2	+	2.08	5.47E−03	0.06	↓	↑
	Hexadecanedioic acid	+	2.06	3.38E−03	0.09	↓	↑
	PI(14:1(9Z)/0:0)	+	2.02	1.99E−03	0.05	↓	↑
	Chlordecone	+	1.99	5.34E−03	0.05	↓	↑
	Prostaglandin B2	+	1.67	2.14E−02	0.03	↓	↑
	PE(P-19:1(12Z)/0:0)	+	1.63	8.75E−04	0.13	↓	↑
	GDP-glucose	+	1.63	1.30E−03	0.02	↓	↑
	6-Keto-prostaglandin F1a	+	1.62	3.48E−02	0.05	↓	↑
	TG(15:0/16:0/22:4(7Z,10Z,13Z,16Z))	+	1.56	1.85E−04	7.7	↑	↓
	Alpha-Linolenoyl ethanolamide	+	1.56	1.25E−06	0.04	↓	↑
	PC(10:0/10:0)	+	1.52	6.89E−08	99.1	↑	↓
	Glycocholic acid	−	1.99	2.02E−02	7.55	↓	↑
	Alanyltryptophan	−	1.94	1.80E−04	0.11	↑	↓
	Propionylcarnitine	−	1.9	1.21E−03	12.4	↓	↑
	Sphingosine 1-phosphate	−	1.84	3.84E−05	0.03	↑	↓
	PI(18:1(9Z)/0:0)	−	1.81	2.62E−09	0	↑	↓
	LysoPC(18:0)	−	1.75	1.55E−06	0.06	↑	↓
	Gamma-glutamylglutamic acid	−	1.73	6.41E−04	6.41	↓	↑
	TG(21:0/22:0/22:1(13Z))	−	1.73	1.58E−07	0.08	↑	↓
	PS(18:3(6Z,9Z,12Z)/0:0)	−	1.66	1.29E−06	0.14	↑	↓
	Angiotensin IV	−	1.62	1.63E−04	0.05	↑	↓
	4-Aminohippuric acid	−	1.57	2.39E−07	0.11	↑	↓
	LysoPE(0:0/15:0)	−	1.54	1.61E−07	0.06	↑	↓
	DG(18:1(11Z)/22:2(13Z,16Z)/0:0)	−	1.52	1.70E−03	7.68	↓	↑
	Estrone sulfate	−	1.52	2.40E−02	0.03	↑	↓
	Phenoxybenzamine	−	1.51	3.11E−07	0.09	↓	↓
	PI(22:6(4Z,7Z,10Z,13Z,16Z,19Z)/0:0)	−	1.78	5.35E−10	0.02	↑	↓
	2-Amino-4-oxo-4-alpha-hydroxy-6-(erythro-1ʹ,2ʹ,3ʹ-trihydroxypropyl)-5,6,7,8-tetrahydroxypterin (A)	−	2.08	3.74E−05	13.67	↓	↑

In the positive ion mode, 11 and 9 metabolites were upregulated and downregulated, respectively. In the negative ion mode, 6 and 3 metabolites were upregulated and downregulated in the urine of the UIRI group, respectively. Among these, 16 metabolites in the positive ion mode and 6 metabolites in the negative ion mode were recovered after the administration of rhein, and in total, 25 metabolites were recovered (Fig. 6D). PCA and PLS-DA for the 25 differential metabolites (Fig. 6E) showed similar changes in the metabolic profiles to Fig. 6B, indicating that the differential metabolite classification results were reliable.

#### Metabolomic analysis of serum samples

**Fig. 7:**
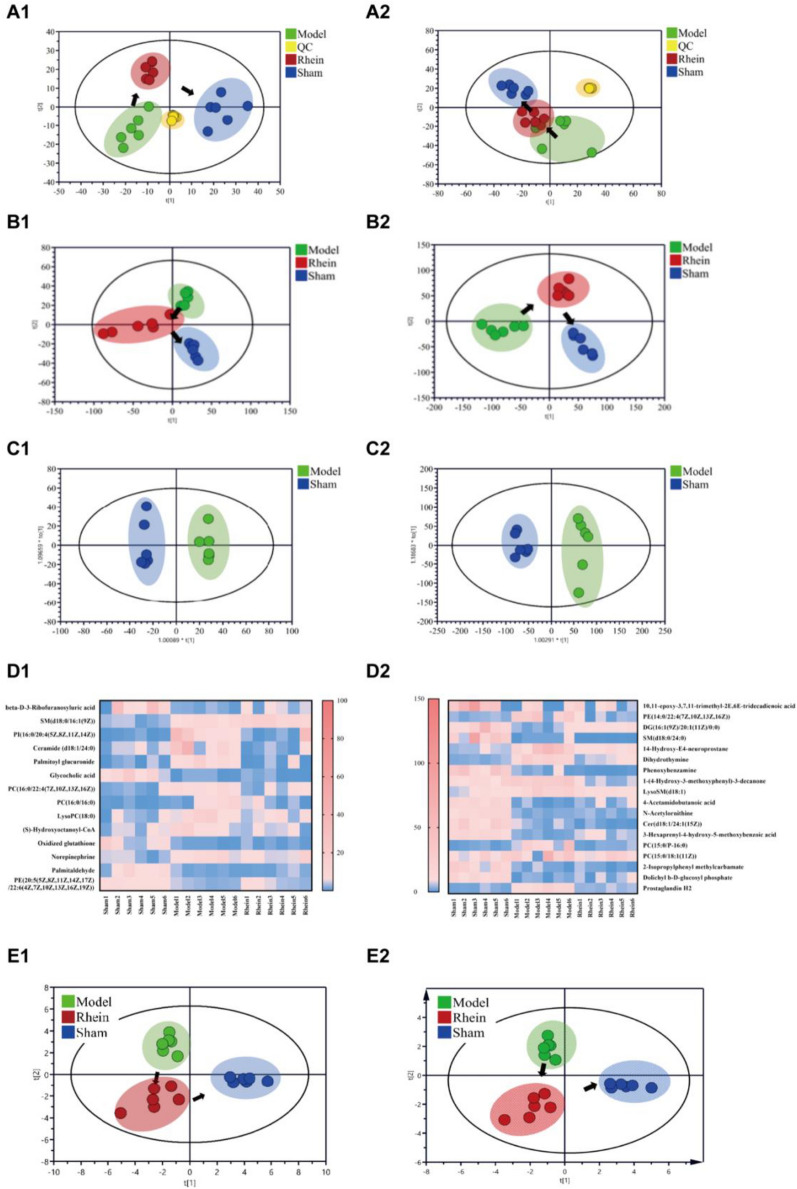
Metabolomics analysis of serum samples in the positive (1) and negative (2) model. **A** PCA score plot; **B** PLS score plot; **C** OPLA-DA score plots of control and TGF-β induction groups; **D** heatmap of relative peak areas of differential metabolites in different groups. PCA (**E1**) and PLS-DA (**E2**) scores for different groups of serum samples based on differential metabolites

Additional file 1: Figure S3 depicts the metabolic profiles of all samples in positive and negative ion modes. A total of 1714 ion peaks in positive ion mode and 2860 ion peaks in negative ion mode were identified. As shown in the PCA score plots in Fig. 7, the QCs clustered well, suggesting good reproducibility and precision of the metabolomic method. As shown in the PLS score plots in Fig. 7B, the UIRI, the sham operation, and the rhein administration groups were clearly differentiated, showing that the metabolic profiles differed significantly among the groups. Based on metabolic profiles, the rhein administration group obviously deviated from the UIRI group and approached the sham operation group, indicating that rhein treatment could effectively delayed the progression of CKD.

As shown in the OPLS-DA score plots in Fig. 7C, the UIRI group was obviously distinguished from the sham operation group, and the model evaluation parameters R2Y and Q2 were both close to 1.0 (in positive ion mode: R2Y = 0.992 and Q2 = 0.835; in negative ion mode: R2Y = 0.981 and Q2 = 0.879). In total, 14 differential metabolites in positive ion mode and 18 differential metabolites in negative ion mode were obtained, respectively (Table 2). Figure 7D shows that after model establishment, the levels of 9 metabolites were up-regulated and 5 metabolites were down-regulated in positive ion mode, while 8 metabolites were up-regulated and 10 metabolites were down-regulated in the negative ion mode. Among these, 10 metabolites in positive ion mode and 13 metabolites in negative ion mode were recovered after the administration of rhein. PCA and PLS-DA for these differential metabolites (Fig. 7E) showed similar changes in the metabolic profile to Fig. 7B, indicating the reliability of this differential metabolite classification.

#### Metabolomic of tissue samples

**Fig. 8:**
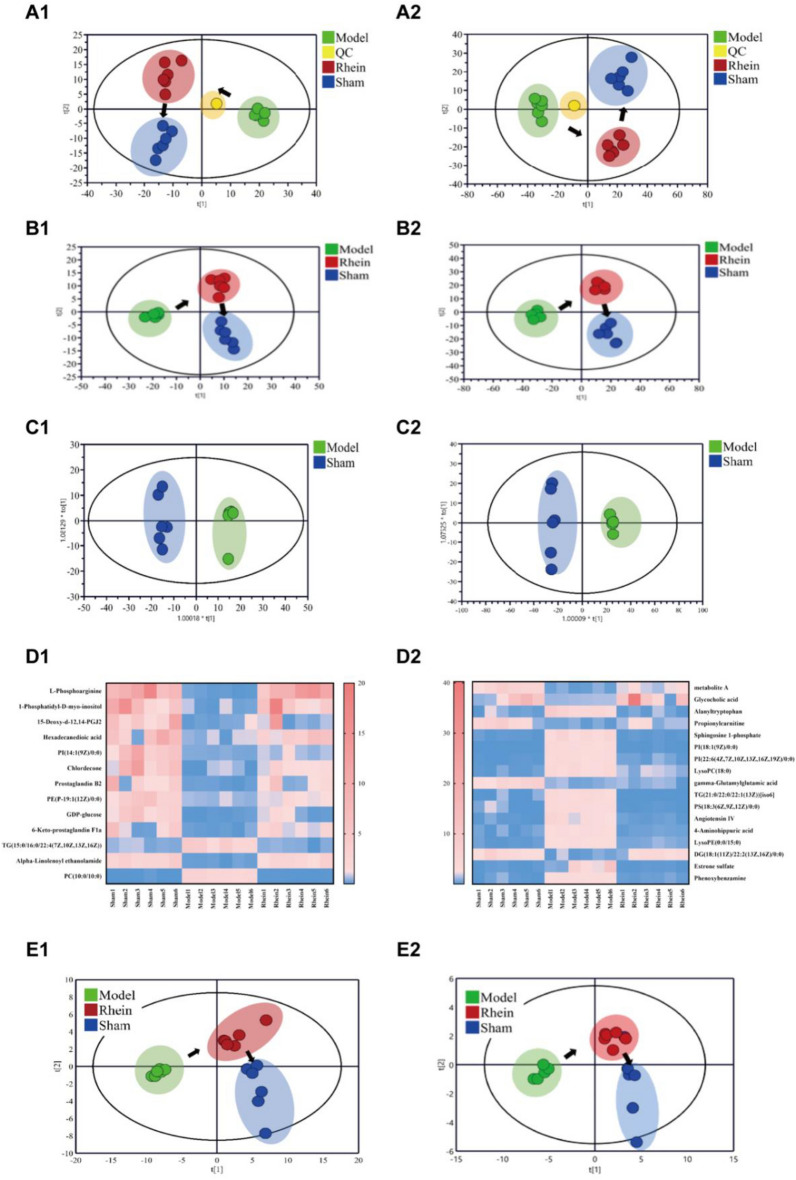
Metabolomics analysis of tissue samples in the positive (1) and negative (2) model. **A** PCA score plot; **B** PLS score plot; **C** OPLA-DA score plots of control and TGF-β induction groups; **D** heatmap of relative peak areas of differential metabolites in different groups. PCA (**E1**) and PLS-DA (**E2**) scores for different groups of tissue samples based on differential metabolites

Additional file 1: Figure S4 depicts the metabolic profiles of all tissue samples in positive and negative ion modes. A total of 539 ion peaks in the positive ion mode and 1415 ion peaks in negative ion mode were identified. As shown in the PCA score plots in Fig. 8A, the QCs clustered well, suggesting good reproducibility and precision of the metabolomic method. As shown in the PLS-DA score in Fig. 8B, the UIRI, sham operation, and rhein administration groups were significantly different, indicating that the metabolic profiles differed among the groups. The rhein administration group obviously deviated from the UIRI group and approached the sham operation group, suggesting that rhein administration delayed renal interstitial fibrosis. As shown in Fig. 8C, in the OPLS-DA score plots, the UIRI group was obviously distinguished from the sham operation group, and the model evaluation parameters R2Y and Q2 were both close to 1.0 (in positive ion mode: R2Y = 0.996 and Q2 = 0.980; in negative ion mode: R2Y = 0.998 and Q2 = 0.968). Multivariate statistical analysis showed that 13 differential metabolites were obtained in positive ion mode, and 17 differential metabolites were obtained in negative ion mode (Table 2). As shown in Fig. 8D, 2 metabolites were up-regulated and 11 metabolites were down-regulated in the positive ion mode; 12 metabolites were up-regulated and five metabolites were down-regulated in the negative ion mode. All of these above metabolites were recovered by rhein. The PCA and PLS-DA results for the differential metabolites are shown in Fig. 8E, showed similar changes in the metabolic profile were similar to the results in Fig. 8B, confirming the reliability of the classification of the screened differential metabolites.

### Network pharmacology and molecular docking of rhein

**Fig. 9:**
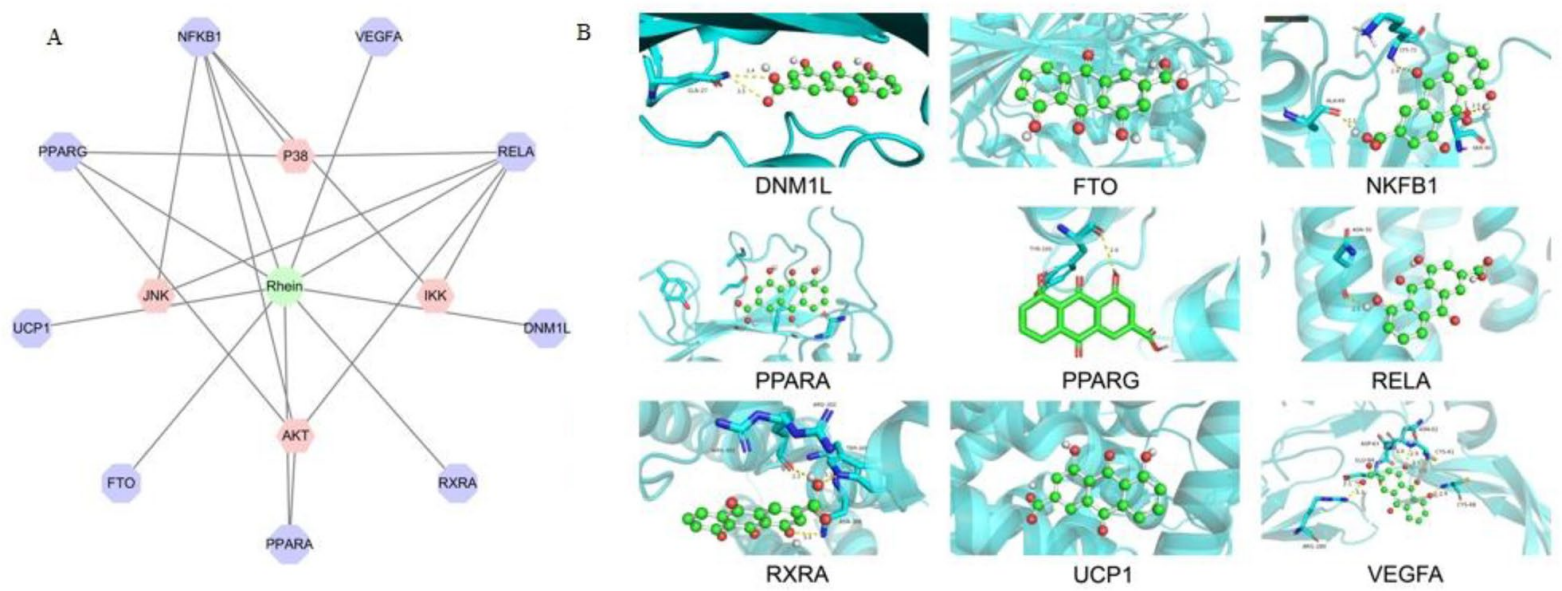
Rhein—target interaction (PPI) network (**A**). Molecular interaction between rhein and its potential targets were performed by docking software (**B**)

Based on the Stitch database, the predicted targets of rhein were NFKB1, RELA, PPARA, PPARG, VEGFA, UCP1, FTO, RXRA, and DNM1L. The interactions between rhein and these targets are shown in Fig. 9. The docking scores of rhein with these targets were all less than − 5 kJ/mol, indicating that rhein had good binding with these targets (Additional file 2: Table S1). The interaction relationship between the targets of rhein and proteins P65, IKK, AKT, P38, JNK, and AP-1 is shown in Fig. 9. NFKB1, RELA, PPARA, and PPARG interacted with AKT, IKK, P38, and JNK, which indicated that rhein acted on the four targets and regulated NF-κB and MAPK signaling pathways.

## Discussion

Currently, there is a lack of targeted and specific therapeutic drugs are lacking. Traditional Chinese medicines can be effective for the treatment of CKD [25]. Rhein exhibited good antifibrotic effects, and delayed the progression of RIF. In this study, we constructed a rat model of renal ischemia–reperfusion fibrosis and a model of cellular fibrosis induced by TGF-β to explore the efficacy and mechanism of action of rhein in delaying renal fibrosis. According to the results presented in Tables 1 and 2, Metabolomics analyses of cells, and rat urine, serum, and tissues showed that there were common or similar differential metabolites between the control, model, and rhein treatment group. These metabolites mainly included phosphatidylcholines (PC), lysophosphatidylcholines (LysoPC), phosphatidylethanolamines, lysophosphatidylethanolamines (LysoPE), and ceramides. They were mainly enriched in sphingolipid metabolism and glycerophospholipid metabolism. There were various types of phosphatidylcholines present in cells and rat urine, serum, and kidney tissue, with the most abundant being found in cells. Lysophosphatidylcholines were also detected in cells and rat urine, serum, and kidney tissue, with LysoPC (18:0) present in both serum and tissue. PE mainly existed in serum and renal tissue, whereas LysoPE was present in cells and rat renal tissue, and neither PE nor LysoPE was detected in urine. Ceramides were mainly present in serum and were not detected in cells, urine, or renal tissues. Hexadecanedioic acid existed simultaneously in urine and tissues, whereas glycocholic acid and phenoxybenzamine coexisted in serum and tissues. Many metabolites in the urine, serum, and kidney tissues, except for PC and LysoPC, were not detected in most cells, indicating a significant difference in metabolism between cells and rats, leading to differences in metabolites in the body. There were also obvious differences in the types of differential metabolites in the serum, urine, and tissues, and the differences in their contents were mainly due to factors such as their source, excretion, composition regulation, and concentration. These differences result from normal physiological processes and help to maintain homeostasis in the internal environment of the human body. Therefore, although analogous metabolites were found in the four sample types, there were only a few exact matches. This suggests that there are significant differences in the differential metabolite contents across different biological samples.

Sphingolipid molecules have multiple effects on cellular metabolism, such as the regulation of energy production and nutrient utilization, and elevated sphingolipid levels are closely associated with renal disease [26, 27]. Merscher et al. [28] studied patients with glomerulosclerosis and diabetic nephropathy and found that the high levels of sphingolipids in glomerular cells can lead to renal insufficiency, and that lipid toxicity caused by sphingolipid dysregulation can cause glomerular damage. Boini et al. [29] found that the mice fed a high-fat diet have increased levels of sphingolipids, ceramides, and their metabolites, which cause insulin resistance and associated glomerular damage. A relationship between sphingolipid-induced kidney damage and inflammatory responses was revealed by Miyamoto et al. [30]. In the kidneys of mice with diabetes and high-fat diet-induced obesity with sphingolipid accumulation, the glomerular ATP/AMP ratio is increased, which inhibits AMPK and causes renal fibrosis. Additionally, studies have shown that ceramide (Cer) metabolites can lead to the aggregation of low-density lipoproteins, produce pro-inflammatory factors, and damage blood vessels [31, 32]. These processes can be catalyzed by platelet ceramide alkaline hydrolase 1 (ACER1) to generate sphingosine. An increase in ACER1 can affect Cer metabolites and generate a large amounts of sphingosine, which can induce vascular inflammation [33, 34]. These findings indicate that increased in sphingosine-1-phosphate and ceramide levels stimulate the occurrence of fibrosis by promoting inflammation. A previous study has reported that regulating the NF-κB and Nrf-2/HO-1 signaling pathways alleviates inflammation and oxidative stress to prevent RIF [35]. A study by Yu et al. [10] showed that rhein downregulates phosphorylated NF-κB, p65, IκBα, and IKKβ induced by LPS and reduces the activation of NF-κB by inhibiting the expression and phosphorylation of related proteins in the NF-κB signaling pathway, thereby inhibiting the transcription of NF-κB and p65; thus, rhein has a protective effect against acute kidney injury. Network pharmacology and molecular docking indicated that rhein directly targeted NF-κB, indirectly regulated AKT and IKK, and inhibited their transcription. The levels of sphingosines, ceramides, and certain proteins (p-P65, p-IKK, and p-AKT) were upregulated in the fibrosis models. Therefore, it is possible that rhein postpones the development of renal fibrosis via its influence on sphingolipid metabolism and the NF-κB signaling pathway.

Certain metabolites exhibited unique properties in various biological samples. For example, 14-hydroxy-E4-neuroprostane E was solely present in serum, whereas 5-HPETE was exclusively found in urine. 14-Hydroxy-E4-neuroprostane E is an unsaturated carboxylic acid consisting of a 20-carbon skeleton with a function similar to that of prostaglandin E2 [36]. Prostaglandin E2 is considered a pro-inflammatory factor [37, 38]. 5-HPETE significantly increased in the levels of diacylglycerol (DAG), which in turn activated protein kinase C (PKC). PKC is involved in the activation of phospholipase A2, leading to the release of AA. A increase in AA is an important cause of inflammation [39]. The AA metabolic pathway is closely associated with inflammation, and elevated levels of related compounds are likely to cause inflammatory damage to the kidneys. In the present study, the levels of 14-hydroxy-E4-neuroprostane E and 5-HPETE increased in different models of fibrosis, and these changes were reversed by the administration of rhein. Therefore, it is speculated that rhein may delay RIF by affecting AA metabolism, thereby inhibiting inflammation.

Two metabolites, phosphatidylcholine and lysophosphatidylcholine, were screened as differential metabolites among the samples, and both indicated an upward trend in the model group and a downward trend after rhein administration. Phosphatidylcholine is a primary supplier of glycerol, fatty acids, choline, and amino alcohols in the body, and can rightly break through the blood–brain barrier to participate in the synthesis of acetylcholine. PC phospholipids are important substances for the maintenance of physiological functions in the human body and can promote blood circulation, regulate serum lipids, and remove peroxides. The level of oxidative stress in the body is also related to the occurrence of diabetic nephropathy [40, 41]. PC-like phospholipids have been identified as biomarkers of chronic glomerulonephritis, chronic renal failure, end-stage renal disease, and diabetic nephropathy [42]. They catalyse the conversion of phosphatidylcholine to lysophosphatidylcholine (Lyso-PC) via phospholipase A2 (PLA2). Lyso-PC levels are significantly increased in hypoalbuminemia nephropathy rats [43]. This compound can also produce lysophosphatidic acid (LPA) in the extracellular fluid after hydrolysis. Excessive formation and secretion of LPA lead to an increase in the AP-1 content. Activation of the downstream MAPK signaling pathway leads to fibroblasts [44]. Rhein can target RELA and PPARG, which regulate JNK, IKK, and P38 proteins, resulting in the activation of the MAPK signaling pathway. Consequently, LPC could trigger this pathway by affecting the inflammatory response and producing LPA, which can lead to fibrosis.

In the pathological mechanism of diabetic nephropathy, the activation of PKC pathway leads to raised phospholipase A2 (PLA2) activity [45]. It is speculated that an increase in phospholipase activity accelerates the hydrolysis of PC to form Lyso-PC, which leads to an increase in the LPC concentration. LPCs are a group of phospholipids and contain only one fatty acid, that can induce the pro-inflammatory mediator cyclooxygenase-2 (COX-2) to participate in the inflammatory response. In our study, PC-like phospholipid levels in the model group induced by TGF-β1 were significantly higher than those in the control group, and rhein decreased the levels of PC-like phospholipids and Lyso-PC significantly. These findings suggested that the disruptions of glycerophospholipid metabolism is an important pathway for the occurrence of cellular fibrosis and that the effects of rhein are mediated by two pathways. Phosphatidylcholine is also involved in the synthesis of 5-HPETE. Though the PLA2 enzyme, phosphatidylcholine generates AA, which then generates 5-HPETE through 5-lipoxygenase (ALOX5) [46]. In summary, rhein reduced the levels of lysophospholipids and phosphatidylcholine, and thus inhibited cell proliferation by participating in glycerophospholipid metabolism and linoleic acid metabolism.

## Conclusion

In this study, cell, and rat urine, serum, and tissue metabolomics approaches using UPLC-QTOF-MS were performed to investigate the effects of rhein in the treatment of CKD. Based on this analysis, the disruptions of sphingolipid metabolism, AA metabolism, and glycerophospholipid metabolism contributes to the development of renal fibrosis. Rhein may contribute to AA metabolism and the glycerophospholipid pathway by regulating metabolites, such as 14-hydroxy-E4-neuroprostane E, 5-HPETE, phosphatidylcholine, and lysophosphatidylcholine, thereby affecting the occurrence and development of renal fibrosis. Moreover, rhein may inhibited the MAPK signaling pathways and NF-κB signalling pathway, suggesting that rhein alleviates the inflammatory response and oxidative stress, and can, thus delay chronic nephropathy.

### Supplementary Information

**Additional file 1: Figure S1.** Total ion chromatogram (TIC) of NRK-49F cell samples. A: Sham group; B: 10 ng/mL TGF-β group; C: rhein administration group. **Figure S2.** Total ion chromatogram (TIC) of urine samples in the positive (1) and negative (2) model. A: Sham group; B: 10 ng/mL TGF-β group; C: rhein administration group. **Figure S3.** Total ion chromatogram (TIC) of serum samples in the positive (1) and negative (2) model. A: Sham group; B: 10 ng/mL TGF-β group; C: rhein administration group. **Figure S4.** Total ion chromatogram (TIC) of tissue samples in the positive (1) and negative (2) model. A: Sham group; B: 10 ng/mL TGF-β group; C: rhein administration group. **Figure S5.** A rhein-target-protein network and molecular docking between rhein and targets.

**Additional file 2.** The molecular docking score and interation between rhein and its potential targets.

## Data Availability

The authors declare that all data supporting the findings of this study are available within the article and its uploaded attach files.

## Abbreviations

CKD: Chronic kidney disease
UPLC-QTOF-MS: Ultra-high performance liquid chromatography-quadrupole time-of-flight mass spectrometry
MDA: Malondialdehyde
SOD: Superoxide dismutase
GSH-Px: Glutathione peroxidase
CAT: Catalase
Scr: Serum creatinine
BUN: Blood urea nitrogen
CMC-Na: Carboxymethyl cellulose
UIRI: Unilateral ischemia–reperfusion injury model
HE: Haematoxylin–eosin
ESI: Electrospray ionization source
QC: Quality control samples
RSD: Relative standard deviation
PCA: Principal component analysis
PLS-DA: Partial least squares discriminant analysis
OPLS-DA: Orthogonal partial least squares discriminant analysis
VIP: Variable importance in projection
FC: Fold change
