# Supplementary material for: Cell and rat serum, urine and tissue metabolomics analysis elucidates the key pathway changes associated with chronic nephropathy and reveals the mechanism of action of rhein
Source: Chin Med. 2023 Dec 1;18:158. doi: 10.1186/s13020-023-00862-1 (PMC10691122; doi:10.1186/s13020-023-00862-1)
Supplement: Supplementary file 1 — Additional file 1: Figure S1. Total ion chromatogram (TIC) of NRK-49F cell samples. A: Sham group; B: 10 ng/mL TGF-β group; C: rhein administration group. Figure S2. Total ion chromatogram (TIC) of urine samples in the positive (1) and negative (2) model. A: Sham group; B: 10 ng/mL TGF-β group; C: rhein administration group. Figure S3. Total ion chromatogram (TIC) of serum samples in the positive (1) and negative (2) model. A: Sham group; B: 10 ng/mL TGF-β group; C: rhein administration group. Figure S4. Total ion chromatogram (TIC) of tissue samples in the positive (1) and negative (2) model. A: Sham group; B: 10 ng/mL TGF-β group; C: rhein administration group. Figure S5. A rhein-target-protein network and molecular docking between rhein and targets. [file 13020_2023_862_MOESM1_ESM.docx]

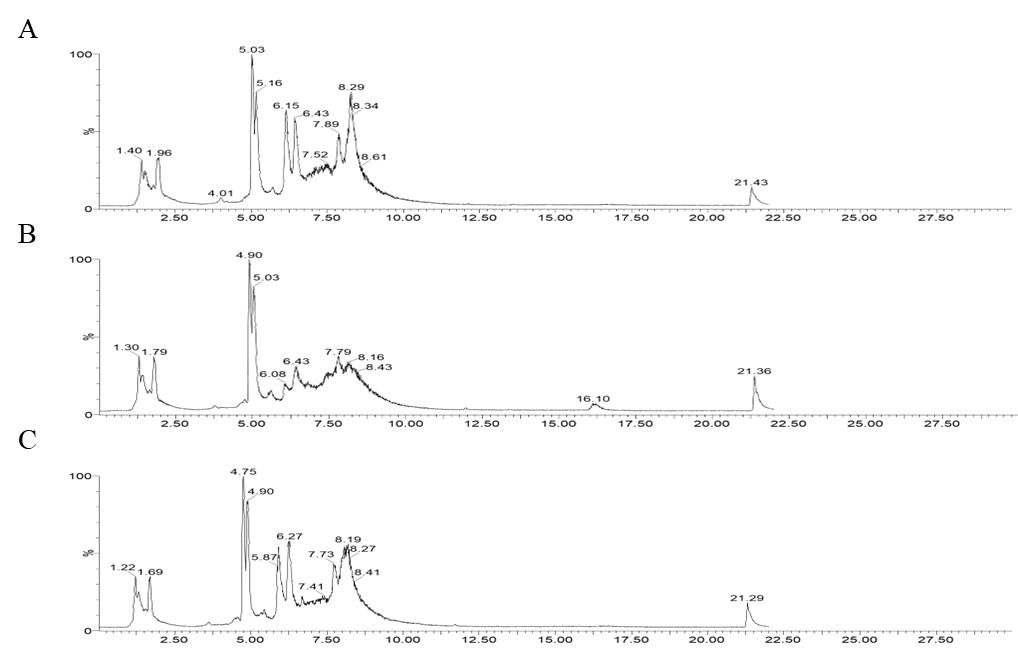


Figure S1 | Total ion chromatogram (TIC) of NRK-49F cell samples. A: Sham group; B: 10 ng/mL TGF-β group; C: rhein administration group.


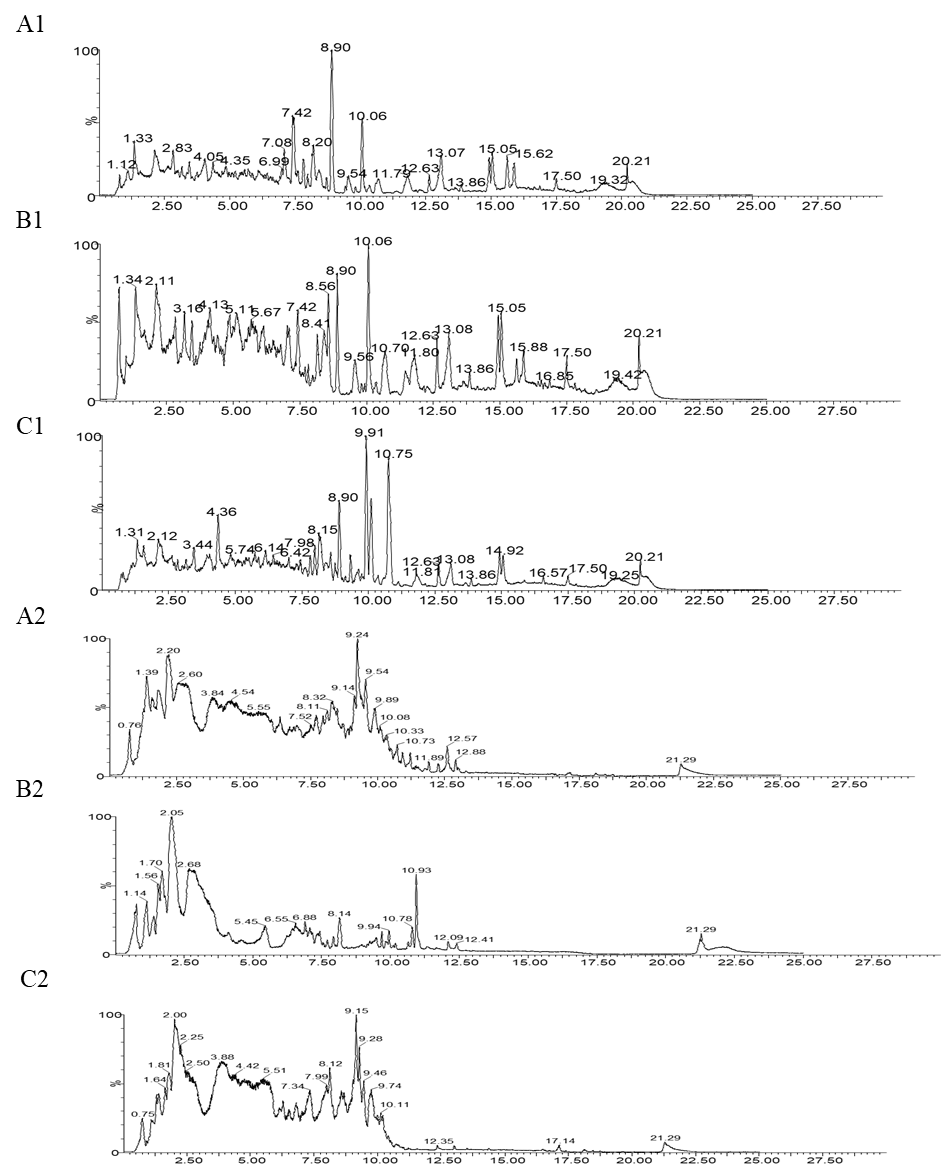


Figure S2 | Total ion chromatogram (TIC) of urine samples in the positive (1) and negative (2) model. A: Sham group; B: 10 ng/mL TGF-β group; C: rhein administration group.


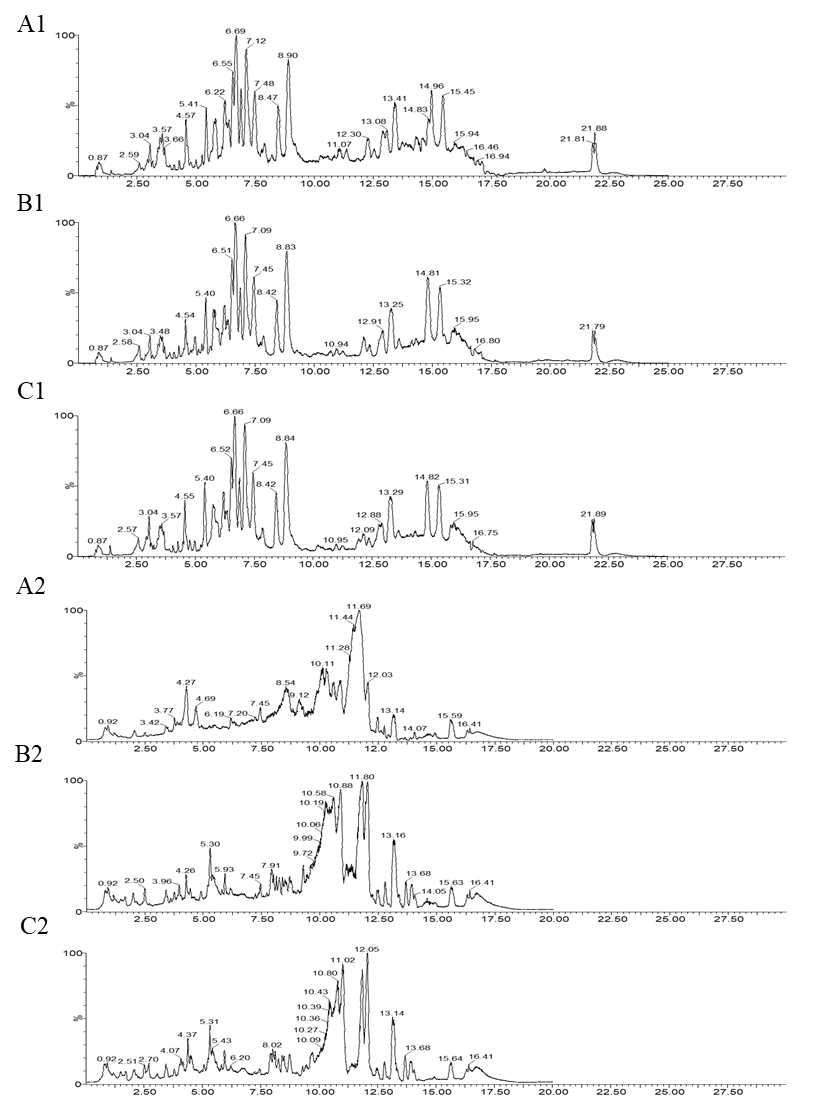


Figure S3 | Total ion chromatogram (TIC) of serum samples in the positive (1) and negative (2) model. A: Sham group; B: 10 ng/mL TGF-β group; C: rhein administration group.


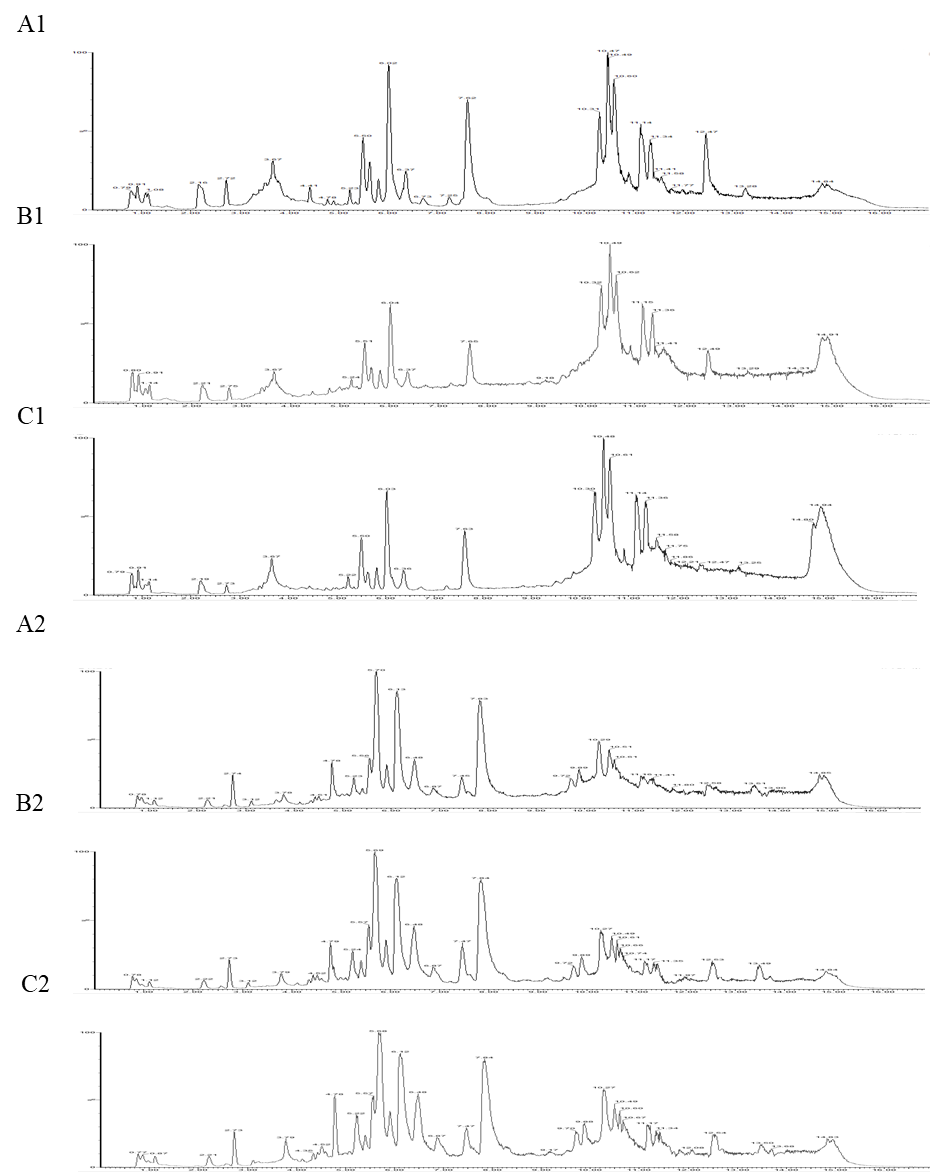


Figure S4 | Total ion chromatogram (TIC) of tissue samples in the positive (1) and negative (2) model. A: Sham group; B: 10 ng/mL TGF-β group; C: rhein administration group.


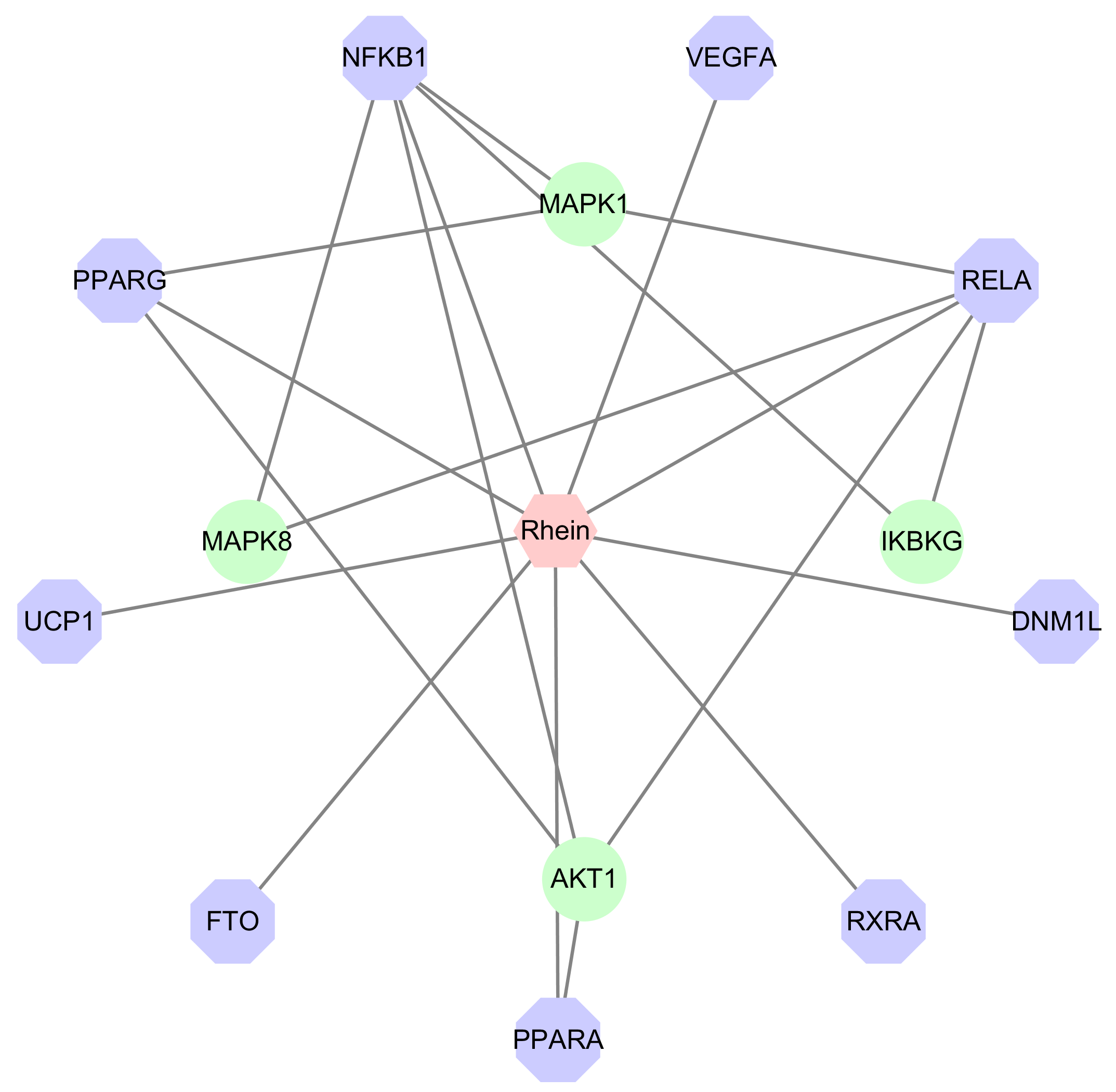


Figure S5 | A rhein-target-protein network and molecular docking between rhein and targets.
